# Adapting SAM2 Model from Natural Images for Tooth Segmentation in Dental Panoramic X-Ray Images

**DOI:** 10.3390/e26121059

**Published:** 2024-12-06

**Authors:** Zifeng Li, Wenzhong Tang, Shijun Gao, Yanyang Wang, Shuai Wang

**Affiliations:** School of Aeronautic Science and Engineering, Beihang University, 37 Xueyuan Road, Haidian District, Beijing 100191, China; lizifeng@buaa.edu.cn (Z.L.); tangwenzhong@buaa.edu.cn (W.T.); gaoshijun@buaa.edu.cn (S.G.); wangyanyang@buaa.edu.cn (Y.W.)

**Keywords:** X-ray, tooth, segmentation, SAM2, deep learning, knowledge distillation, small sample dataset

## Abstract

Dental panoramic X-ray imaging, due to its high cost-effectiveness and low radiation dose, has become a widely used diagnostic tool in dentistry. Accurate tooth segmentation is crucial for lesion analysis and treatment planning, helping dentists to quickly and precisely assess the condition of teeth. However, dental X-ray images often suffer from noise, low contrast, and overlapping anatomical structures, coupled with limited available datasets, leading traditional deep learning models to experience overfitting, which affects generalization ability. In addition, high-precision deep models typically require significant computational resources for inference, making deployment in real-world applications challenging. To address these challenges, this paper proposes a tooth segmentation method based on the pre-trained SAM2 model. We employ adapter modules to fine-tune the SAM2 model and introduce ScConv modules and gated attention mechanisms to enhance the model’s semantic understanding and multi-scale feature extraction capabilities for medical images. In terms of efficiency, we utilize knowledge distillation, using the fine-tuned SAM2 model as the teacher model for distilling knowledge to a smaller model named LightUNet. Experimental results on the UFBA-UESC dataset show that, in terms of performance, our model significantly outperforms the traditional UNet model in multiple metrics such as IoU, effectively improving segmentation accuracy and model robustness, particularly with limited sample datasets. In terms of efficiency, LightUNet achieves comparable performance to UNet, but with only 1.6% of its parameters and 24.0% of the inference time, demonstrating its feasibility for deployment on edge devices.

## 1. Introduction

Dental panoramic X-ray imaging is a widely used diagnostic method in dentistry due to its cost-effectiveness and low radiation exposure [1]. This imaging technique involves rotating the X-ray source around the patient’s head to capture a comprehensive view of the oral cavity, which is essential for identifying various dental conditions such as tooth decay, periodontal diseases, and structural anomalies. However, the interpretation of these images can be challenging for dentists, primarily due to issues like noise, low edge contrast, and anatomical overlaps in the images. These factors often complicate the identification of pathologies, making it reliant on the dentist’s experience and visual judgment [2]. These issues not only increase the dependence on the dentist’s personal experience but also raise the risk of misdiagnosis and missed diagnosis. Therefore, accurate tooth segmentation is crucial for improving the accuracy and efficiency of diagnosis.

Tooth segmentation provides necessary image information for further lesion analysis and treatment planning by accurately separating the teeth from the background and adjacent structures. This process helps dentists quickly and accurately identify the specific condition of the teeth, such as the location of cavities, the degree of wear, and structural abnormalities [3,4,5]. The application of automatic tooth segmentation technology can significantly reduce the workload of dentists, allowing them to focus more on developing treatment strategies and managing diseases, rather than spending a large amount of time on basic image analysis.

Panoramic dental X-ray images present significant challenges for automatic image analysis due to their coverage of multiple body parts, such as teeth, gums, jaw, skull, spine, and other skeletal structures [6]. Moreover, these images are often affected by low contrast and high noise, making traditional manual feature extraction methods difficult to apply. Before 2018, most research on dental X-ray image analysis relied on manual feature extractors, especially neglecting the analysis of panoramic X-rays, possibly due to the high complexity and difficulties in handling the diverse features of these images [3].

With the rapid development of deep learning technology, its application in medical image analysis has shown great potential and significant advantages [7,8,9,10,11,12]. Especially in image segmentation tasks, deep learning models, particularly convolutional neural networks (CNNs), have become a key technology driving precise and automated medical diagnosis. These models, by learning complex patterns and features from large amounts of labeled data, can automatically identify and segment relevant structures in images, greatly improving the accuracy and efficiency of segmentation tasks.

However, in the research and application of tooth segmentation, obtaining a large number of high-quality dental X-ray images is often a challenge [13]. The collection of these images is limited by various factors, including patient privacy protection, the cost and time of data collection, and the resource consumption of data annotation. Therefore, with the issue of limited data, traditional deep learning model training may face difficulties such as model overfitting and poor generalization ability. Additionally, in the real scenario, deploying traditional deep learning models directly on edge devices, such as X-ray machines, is challenging due to their limited computational resources and memory capacity. These models, with their large number of parameters and high memory demands, make it difficult to perform inference locally. As a result, using such models on edge devices without relying on cloud computing becomes impractical, which limits their applicability in many practical medical environments.

In summary, applying deep learning models to dental X-ray image datasets faces two main challenges: first, the limited amount of data often leads to overfitting during training, which restricts the model’s generalization ability; second, existing high-precision deep models typically require a large number of parameters and computational resources, making efficient deployment in practical systems highly challenging.

**Figure 1:**
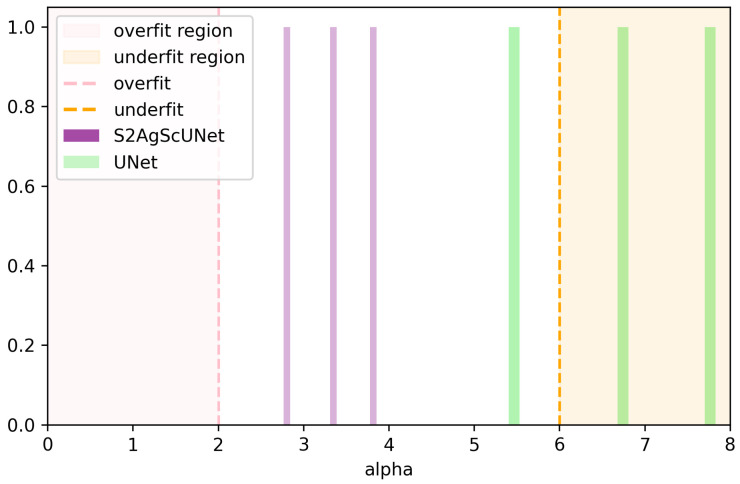
Analysis and comparison of the weights of the UNet and S2AgScUNet models. According to the HSTR theory [22], the alpha value indicates the quality of training for each layer, with values between 2 and 6 representing well-trained layers. A value greater than 6 suggests overfitting, while a value below 2 implies underfitting. Two components of the UNet model have alpha values exceeding 6, indicating a high risk of overfitting.

**Figure 2:**
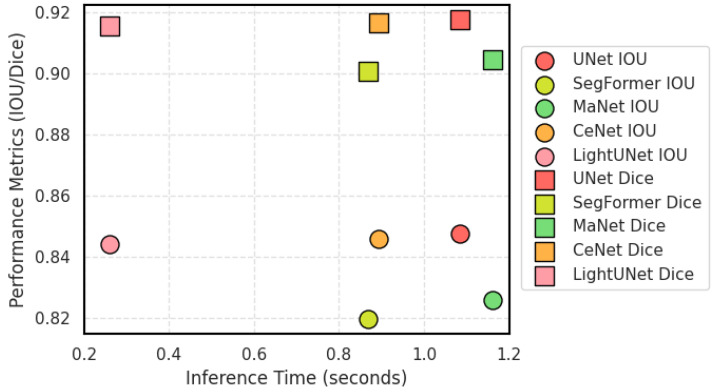
Comparison of inference time and performance. The horizontal axis represents the inference time (seconds), while the vertical axis shows performance metrics (IOU and Dice). LightUNet significantly outperforms other models in inference time while achieving performance comparable to or better than other models.

For the first challenge, using pre-trained large models, such as SAM [14], SAM2 [15], becomes an efficient solution. The SAM2 model, pre-trained on large-scale general image datasets, has already acquired a rich capability for image feature representation. This allows it to effectively extract key features, even when the dental X-ray image data is limited, enabling accurate tooth recognition and segmentation. As a result, scholars have made numerous attempts to apply SAM to medical image analysis, achieving encouraging results. These studies not only demonstrate the efficiency of SAM in handling complex medical images but also validate its great potential for application in the medical field [16,17,18,19,20,21]. To specifically illustrate our motivations, we introduce an evaluation metric, alpha, based on the theory of Charles H. Martin and others [22], which suggests that model performance can be evaluated through the analysis of model weights without relying on data. If alpha as a value lies between 2 and 6, it indicates good model performance. In contrast, a value above 6 suggests potential overfitting, while a value below 2 indicates underfitting. Figure 1 displays the alpha values for the various components of the UNet and the S2AgScUNet model. The S2AgScUNet model is our proposed model, which includes the Encoder part of the pre-trained SAM2 model. Notably, two components of the UNet have alpha values exceeding 6, indicating potential overfitting. In contrast, all three components of the S2AgScUNet fall within the range of 2 to 6. This demonstrates that the introduction of SAM2 effectively alleviates overfitting issues associated with small sample datasets in the dental segmentation field. Additionally, we used the S2AgScUNet model as a teacher model and employed knowledge distillation to construct a lightweight model suitable for deployment on edge devices, called LightUNet. In Figure 2, we show the inference time and accuracy of LightUNet compared to other models. As seen in the figure, LightUNet significantly outperforms other models in inference time while achieving performance comparable to or better than the traditional UNet and other complex models.

As a result, we summarize our methodology to address the two issues:

- We adopt a fine-tuning approach based on a pretrained model. While some researchers have attempted to transform SAM into a U-shaped architecture [20,23], these efforts were limited by the simple structure of the standard ViT encoder [24], which lacks the hierarchy required for more complex segmentation tasks. The introduction of SAM2 offers new opportunities for designing more efficient U-shaped networks [25]. Direct application to medical images is less effective, mainly due to the significant differences in structure and features between medical and natural images, such as the low contrast and complex anatomical structures of X-ray images. Therefore, relying solely on the SAM2 model pretrained on natural images is insufficient for medical image segmentation. To address this, we used adapter modules for fine-tuning and introduced feature fusion and selection modules to enhance the model’s semantic understanding and multi-scale detail capture for medical images. Specifically, we added adapter modules to the Encoder part of the SAM2 model and used it as the Encoder part of our model. Then, ScConv [26] modules were introduced before the skip connections to reduce redundancy in feature extraction. We also applied gated attention in the skip connections to further enhance detail segmentation, resulting in a model suitable for small-sample dental segmentation datasets, named S2AgScUNet. Experimentally, on the UFBA-UESC dataset [3], the S2AgScUNet model achieved an IoU score of 0.8612, surpassing the 0.8477 achieved by the UNet model.
- We employ a knowledge distillation approach to create a more efficient model suitable for practical deployment. We use the fine-tuned model as the teacher model and then create a lightweight model called LightUNet, which has the same architecture as the UNet model but only 0.016 times its parameters. The inference time on the entire test set is only 0.24 times that of UNet.

In conclusion, this work mainly makes the following contributions:

- **Proposed S2AgScUNet based on the pre-trained SAM2 model**: This paper introduces S2AgScUNet, which leverages the pre-trained SAM2 model as an encoder, combined with ScConv modules and gated attention mechanisms, providing an effective solution for dental panoramic X-ray image segmentation under limited sample conditions.
- **Effectively alleviated the overfitting problem in tooth segmentation**: By incorporating the pre-trained SAM2 model with hierarchical feature learning capabilities, the proposed method significantly reduces overfitting in dental X-ray small-sample datasets, enhancing both the model’s generalization ability and robustness.
- **Significant improvement in segmentation performance**: Experimental results on the UFBA-UESC dataset demonstrate that the S2AgScUNet model significantly outperforms the traditional UNet model in multiple metrics, such as IoU and Dice, particularly excelling in capturing details and segmenting complex structures.
- **Efficient deployment on edge devices using knowledge distillation**: To facilitate deployment on edge devices, we utilized knowledge distillation to achieve comparable performance to the UNet model, while reducing the parameter count to only 0.49 million compared to UNet’s 31.05 million parameters, making our model more efficient and lightweight.

## 2. Related Work

### 2.1. Classical Statistical Methods

Various classical methods have been proposed in the past decades, but efficacy was limited by the weak image quality and the inconspicuous boundaries of teeth. Region-based segmentation methods, such as those used by Lurie et al. [27] for panoramic X-ray images to aid in osteopenia and osteoporosis detection, and Modi and Desai [28] with a region growing approach for bitewing X-ray images, focus on dividing images into regions based on pixel intensity discontinuities. Radhiyah and Harsono [29] preprocessed panoramic X-ray images by utilizing Gaussian and histogram equalization filters. Meanwhile, Alsmadi [30] and Son et al. [31] utilized clustering to perform the segmentation of tooth X-ray images. However, the methods by Lurie et al. and Modi and Desai did not leverage boundary information, which was later addressed by Ali [32] and Ejbali [33], who utilized active contours to effectively delineate between teeth and background in cases of weak gradient prominence. Li [34] and Fevens [35] developed two boundary strategies that integrate level sets with support vector machines (SVM), effectively addressing the challenge of defining initial contours for coupled level sets. Additionally, several machine learning approaches that are resilient to missing values, such as decision trees (DT) and random forests (RF), tend to have high time complexity due to their reliance on greedy search strategies [36,37]. In some instances, it becomes necessary to account for the gray levels of pixels associated with the target object, which are fundamentally distinct from the background. To address this issue, threshold methods were introduced [38]. However, substantial variations in pixel intensity related to dominant objects can introduce new challenges. To tackle the problem of uneven pixel intensity distribution with global thresholds, adaptive thresholding methods [39] were proposed for improved results.

### 2.2. Deep Learning-Based Methods

Deep neural networks leverage data to support various medical applications. Unlike traditional tooth segmentation methods that rely on complex rules for modeling, data-driven deep learning approaches offer superior modeling power and better generalization capabilities [40,41,42,43,44,45,46,47]. Tekin et al. [48] applied Mask R-CNN to segment and label teeth in occlusal radiography images, achieving high-quality segmentation masks. Yang et al. [49] developed a more streamlined and automated method for dental image analysis that incorporates dental diagnostic knowledge, significantly reducing manual effort in data preparation. Furthermore, Xia et al. [50] introduced upper and lower mandibles from CT scans into their model, successfully isolating a single tooth and creating a complete model of it from CT images where upper and lower teeth naturally contact. The UNet-based network provides a new research idea for recent teeth segmentation tasks. Koch et al. [51] implemented semantic segmentation of panoramic dental images using the UNet network, which achieves better sample segmentation with a smaller, simpler network structure. Kong et al. [52] designed the Efficient Encoder-Decoder Network (EED-Net) for quick and precise segmentation of maxillofacial regions, featuring a residual encoder, a multipath feature extractor, and an object-oriented decoder. Additionally, Zhao et al. [53] introduced the two-stage attention segmentation network (TSASNet) for tooth localization and segmentation in dental panoramic X-ray images, which effectively gathers pixel-level context information and identifies unclear tooth regions. UNet architectures have demonstrated strong performance in medical image segmentation tasks. Numerous researchers have built on the original UNet structure, focusing on enhancements such as refining the encoder and decoder, optimizing convolutional layers, and advancing skip connection techniques [5,54,55,56].

Despite advancements, the effectiveness of deep learning models remains limited by the quality and volume of data [40]. Current information indicates that the largest X-ray dental segmentation dataset contains only 1500 images, spread across 10 categories—with each category containing merely 150 images [3]. This small dataset size is inadequate for effectively training deep learning models, raising concerns such as overfitting. Such overfitting can significantly degrade the model’s ability to generalize, resulting in poor performance when the model encounters new, unseen data. This further underscores the necessity of incorporating pretrained large models in the field of dental segmentation for X-ray images. Such models can leverage learned features from extensive datasets, mitigating issues arising from limited data availability.

## 3. Method

### 3.1. Model Architecture

**Figure 3:**
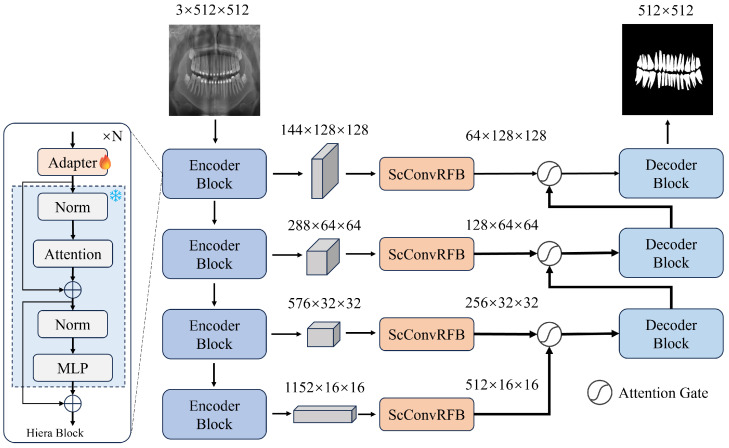
Structure of S2AgScUNet model. The Encoder Block consists of an adapter with gradients and a non-gradient Hiera Block [57], detailed in Section 2.1.1. The ScConvRFB module is used to enhance feature representation and reduce redundancy, as described in detail in Section 2.1.2. The Attention Gate is employed to focus on relevant features during upsampling, enhancing the precision of segmentation, as detailed in Section 2.1.3. The Decoder Block is responsible for gradually upsampling the feature maps while incorporating skip connections to refine the segmentation output, as explained in detail in Section 2.1.4.

The overall architecture of S2AgScUNet is shown in Figure 3, consisting of five main components: Encoder, Decoder, Receptive Field Block (RFB), Adapter, and Gated Attention module (Ag). It is important to note that we only used the image encoder part from the SAM2 model.

#### 3.1.1. Semantic Medical Adapter for Fast Tuning

Due to the complexity and difficulty in acquiring standard X-ray images for dental segmentation, datasets with X-rays are relatively scarce, with the largest dataset containing only 1500 images. Directly applying deep learning models on such small datasets can lead to overfitting. Introducing pretrained models can effectively mitigate this issue, as demonstrated in Figure 1.

S2AgScUNet utilizes the Hiera [57] backbone pretrained by SAM2. Compared to the standard ViT [24] encoder used in SAM, Hiera employs a hierarchical structure that enables better multi-scale feature capture, making it more suitable for designing U-shaped networks. Specifically, given an input image $I\in R^{3\times H\times W}$, where *H* represents the height and *W* represents the width, Hiera outputs four hierarchical features:

$$X_{i}\in R^{C_{i}\times \frac{H}{2^{i+1}}\times \frac{W}{2^{i+1}}},\;\left(i\in \left\{1,2,3,4\right\}\right)$$

For Hiera-L, $C_{i}\in \left\{144,288,576,1152\right\}$.

Due to the large number of parameters in Hiera (e.g., 214 M for Hiera-L), fully fine-tuning is not always feasible in terms of memory usage. To enable efficient fine-tuning, we froze Hiera’s parameters and inserted adapters before each multi-scale block. Similar to the Adapter designs in [21], each Adapter in our framework consists of a linear layer for downsampling, a GeLU activation function, another linear layer for upsampling, and a final GeLU activation function. The structure can be represented as follows:

(1) $$\mathrm{Adapter}\left(X\right)=\mathrm{GeLU}({\mathrm{Linear}}_{\mathrm{up}}\left(\mathrm{GeLU}\left({\mathrm{Linear}}_{\mathrm{down}}\left(X\right)\right)\right))$$

where ${\mathrm{Linear}}_{\mathrm{down}}$ and ${\mathrm{Linear}}_{\mathrm{up}}$ are linear layers for downsampling and upsampling, respectively, and GeLU is the activation function.

#### 3.1.2. Receptive Field Block for Feature Enhancement and Redundancy Reduction

Although the model was originally pretrained on natural images, X-ray images fundamentally differ from natural images. Thus, even with the integration of an Adapter for fine-tuning, it is challenging to completely avoid redundancy and the emergence of noise in the features. To address this issue, Receptive Field Blocks (RFB) are introduced to enhance feature extraction by expanding the model’s receptive field, allowing it to better capture contextual information and reduce irrelevant features. This helps improve the model’s robustness and its ability to focus on relevant structures, which is crucial for accurate segmentation in medical images.

After extracting Encoder features, we pass them through four receptive field blocks [25] to align the channel dimensions with the subsequent Decoder. Then, the ScConv module is introduced to reduce redundancy in spatial and channel features, enhancing effective features while suppressing noise. The module consisting of SRU and CRU. The input features are sequentially processed through the SRU and CRU modules. SRU is used to separate redundant features based on weights and reconstruct them, suppressing redundancy in the spatial dimension and enhancing feature representation. CRU utilizes a split-transform-merge strategy to reduce redundancy in the channel dimension and decrease computational cost and storage requirements.

#### 3.1.3. Attentional Gating for Multi-Scale Fusion

Before the traditional skip connections transfer features from the encoder directly to the corresponding layers of the Decoder, we introduce attention gates to weight these features. This allows the model to focus on more informative regions while ignoring the background and irrelevant areas. The attention mechanism uses a sigmoid activation function to generate weight maps, which are multiplied by the output of the encoder to concentrate on critical features, thereby enhancing the accuracy of detail segmentation.

**Figure 4:**
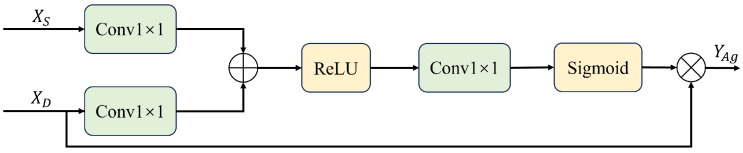
Structure of the Gated Attention module. $X_{S}$ denotes the input from the skip connection, representing features from the corresponding encoder layer, while $X_{D}$ represents the features from the Decoder layer. The Conv1×1 blocks are convolution layers with a filter size of 1×1, used to refine the features. ReLU is the Rectified Linear Unit activation function, introducing non-linearity into the network, and Sigmoid is the activation function that generates the weight map for gating.

The structure and processing flow of the Gated Attention module are shown in Figure 4. It takes two inputs, $X_{S}$ and $X_{D}$, where $X_{S}$ represents the output of the skip connection from the corresponding layer, and $X_{D}$ represents the output from the Decoder layer. The Gated Attention can be expressed as:

(2) $$Y_{A}g=\sigma \left(W_{R}ReLu\left(W_{S}X_{S}+W_{D}X_{D}\right)\right)⊙X_{D}$$

where $W_{S}$, $W_{D}$ and $W_{R}$ are learnable weights, σ is the sigmoid activation function, ReLu is the ReLU activation function, and ⊙ represents element-wise multiplication. The output $Y_{A}g$ enhances the Decoder features based on the skip connection information.

#### 3.1.4. Decoder

**Figure 5:**
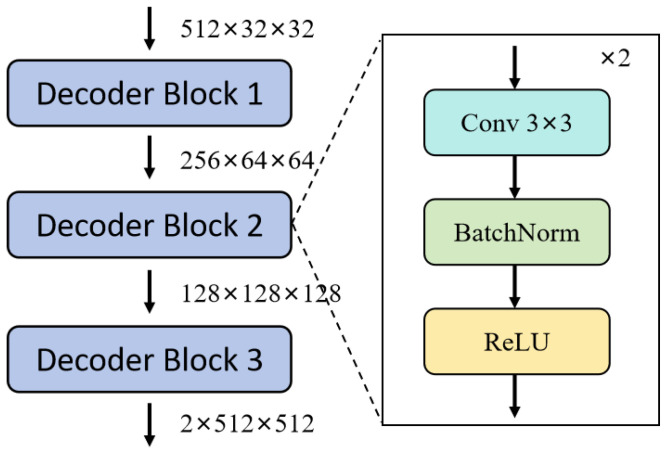
Structure of Decoder Blocks in S2AgScUNet. Each Decoder Block consists of two basic blocks, which include convolution, BatchNorm, and ReLU layers, aiming to reconstruct spatial details for segmentation.

The configuration of the Decoder is identical to that of the UNet model, consisting of four layers of basic convolution blocks. This can be expressed mathematically as follows:

(3) Decoder Block(X)=ReLU(BN(Conv(ReLU(BN(Conv(X))))))

where Conv refers to the convolutional layer, which processes the input feature map with learned filters to extract specific features; BN stands for Batch Normalization, which normalizes the activations from the previous layer to stabilize network training and speed up model convergence; ReLU is the Rectified Linear Unit activation function, introducing non-linearity that helps to address the problem of vanishing gradients, thus enhancing training efficiency. Its detailed structure and processing flow are shown in Figure 5.

The Decoder follows an upsampling strategy, where each layer increases the spatial resolution of the feature maps. The Decoder uses transposed convolution (also known as deconvolution) for upsampling while preserving semantic information. Skip connections are used to concatenate feature maps from the corresponding encoder layers with those from the Decoder. These skip connections are crucial, as they allow the Decoder to leverage both low-level and high-level features, thereby improving segmentation accuracy, particularly in handling details and boundaries.

### 3.2. Training

In this subsection, we describe the training process of the models. First, we trained the S2ScAgUNet model by fine-tuning the pre-trained SAM2 model to address overfitting and improve the overall performance and generalization capability of the model. Then, we employed knowledge distillation, using S2ScAgUNet as the teacher model to train the lightweight LightUNet, aiming to reduce model parameters and computational complexity while maintaining good performance.

#### 3.2.1. Fine-Tuning

In order to effectively address the overfitting issues that often arise with small-sample datasets, we employed a fine-tuning approach using a pre-trained SAM2 model. By leveraging a model that has already been trained on large-scale natural image datasets, we are able to provide a strong initialization, which allows the model to better generalize to dental X-ray images even with limited training samples. This approach effectively reduces the risks of overfitting by utilizing features and patterns learned from diverse datasets, which contribute to a more robust feature extraction process.

During the fine-tuning process, adapter modules were introduced, and the gradients of the original SAM2 model were frozen to enable rapid adjustment of the model parameters to the specific characteristics of dental X-ray images, such as low contrast and complex anatomical structures. This allows the model to focus on relevant features for dental segmentation while minimizing sensitivity to noise and irrelevant variations. As a result, fine-tuning not only enhances segmentation accuracy but also improves the model’s robustness, making it more effective at handling variations in dental X-ray images without succumbing to overfitting.

In our fine-tuning process, we utilized cross-entropy loss to guide the optimization of the segmentation model. The goal was to minimize the discrepancy between the predicted segmentation output and the ground truth labels, thus ensuring more accurate feature extraction and precise segmentation. The cross-entropy loss can be expressed as:

(4) $$L_{CE}=-\sum_{i=1}^{N}y_{i}\log\left({\hat{y}}_{i}\right)$$

where $y_{i}$ represents the true label for pixel *i*, and ${\hat{y}}_{i}$ is the predicted probability of that pixel belonging to the foreground. The cross-entropy loss encourages the model to focus on reducing incorrect predictions while effectively learning correct segmentation boundaries, which is crucial for achieving high-quality results in dental X-ray image segmentation.

#### 3.2.2. Knowledge Distillation and Model Lightweighting

To enable efficient deployment on edge devices, we introduced knowledge distillation. Knowledge distillation is a technique where a smaller model (referred to as the “student model”) learns from a more complex model (referred to as the “teacher model”). Through this approach, we developed a significantly reduced-parameter model called LightUNet. The number of parameters in LightUNet is reduced to only 0.49 million compared to the 31.05 million parameters of the UNet model, significantly reducing model complexity while maintaining comparable performance, making it more suitable for deployment on edge devices.

Knowledge distillation enables a lightweight model to learn from a more complex teacher model, maintaining performance while reducing the model size. Given a teacher model and a student model, the goal of knowledge distillation is to minimize the following loss function in the student model:

(5) $$L_{KD}=\alpha \cdot L_{CE}\left(y,{\hat{y}}_{s}\right)+\left(1-\alpha \right)\cdot T^{2}\cdot L_{KL}\left(\sigma \left({\hat{y}}_{t}/T\right),\sigma \left({\hat{y}}_{s}/T\right)\right)$$

where $L_{CE}$ represents the cross-entropy loss function, used to align the student’s prediction $\hat{y}s$ with the ground truth labels *y*; $L_{KL}$ represents the KL divergence loss, used to match the predictions of the teacher model ${\hat{y}}_{t}$ with those of the student model; σ is the Softmax function, *T* is the temperature coefficient used to smooth the prediction distribution, and α is the weight coefficient to balance the contribution of the cross-entropy loss and the KL divergence loss.

**Figure 6:**
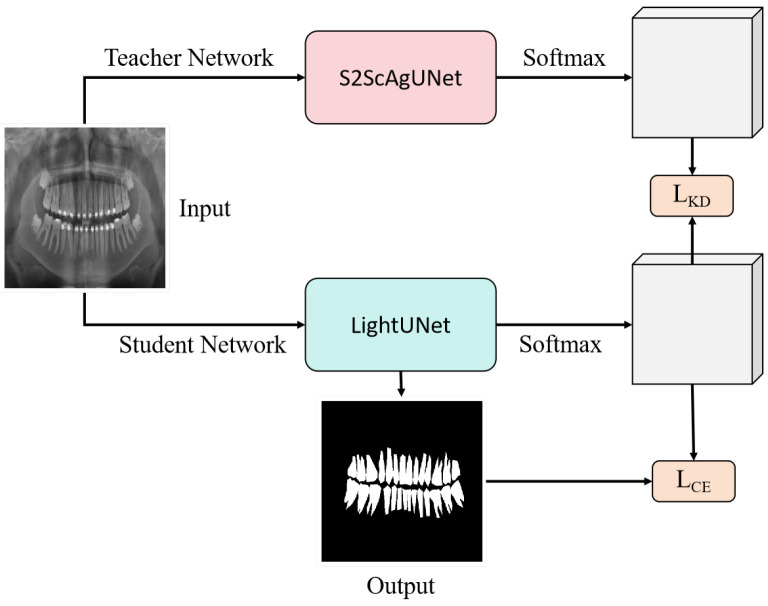
Knowledge distillation workflow based on S2ScAgUNet and LightUNet. The training process consists of two parts: $L_{KD}$, representing the knowledge distillation loss, and $L_{CE}$, representing the cross-entropy loss. The S2ScAgUNet serves as the teacher model, distilling knowledge into LightUNet to reduce parameters while maintaining high performance.

Through this loss function, the student model learns not only from the ground truth labels but also gains additional knowledge from the teacher model, which includes inter-class relationships and richer feature representations. This helps the student model achieve improved performance even with significantly fewer parameters compared to the teacher model. In this work, we use our proposed S2ScAgUNet as the teacher model and LightUNet as the student model, as shown in Figure 6.

## 4. Experiments

### 4.1. Dataset and Experimental Setup

**Dataset.** In dental image analysis, where prediction accuracy is paramount, our preparatory work focuses on carefully selecting a representative dataset and processing it for subsequent model training. Based on the framework established by D. Budagam et al. [58], we selected 425 dental images from the UFBA-UESC dental image dataset proposed by G. Jader et al. [3]. This dataset is a comprehensive collection of anonymous X-ray panoramic dental images characterized by high variability. It contains 1500 images, each sized 512 × 512 × 3, and is categorized into 10 distinct classes, representing various types of dental cases, including the standard 32 teeth, dental instruments, and dental restorations. The dataset also uses images where the number of teeth is less than 32 if they are extracted and more if the jaw has an abnormal mutation. This diversity mirrored the real-world variations in dental scans due to factors such as dental anomalies or missing teeth. The structure of the open data set UFBA-UESC is presented in Table 1.

**Experimental Setup.** To ensure a fair comparison among models, all experiments were conducted on the same GPU with a batch size uniformly set at 16. Adam optimizer was used with an initial learning rate of 0.001, and cross-entropy loss was uniformly applied as the loss function.

### 4.2. Evaluation Metrics

In this study, we used the following metrics to evaluate the performance of the dental segmentation model based on pixel alignment:

**Intersection over Union (IoU)**: IoU measures the ratio of the number of correctly predicted pixels to the total number of pixels that belong to either the predicted or ground truth segment. It is defined as:

(6) $$\mathrm{IoU}=\frac{TP}{TP+FP+FN}$$

where TP (True Positives) is the number of pixels correctly predicted as positive, FP (False Positives) is the number of pixels incorrectly predicted as positive, and FN (False Negatives) is the number of pixels incorrectly predicted as negative. A high IoU indicates that the model accurately distinguishes between teeth and non-teeth areas, which is essential for effective dental diagnostics.

**Dice Coefficient**: The Dice coefficient assesses the overlap between the predicted and ground truth pixels, calculated as:

(7) $$\mathrm{Dice}=\frac{2\times TP}{2\times TP+FP+FN}$$

A Dice value closer to 1 indicates better performance in segmenting the dental images. High Dice scores reflect that the model effectively captures the relevant regions of interest, which is critical for accurate treatment planning. Conversely, low Dice scores may indicate that the model misses significant portions of dental structures.

**Precision**: Precision measures the proportion of correctly predicted positive pixels to the total predicted positive pixels, defined as:

(8) $$\mathrm{Precision}=\frac{TP}{TP+FP}$$

High precision indicates that the model has fewer false positives. In the context of dental segmentation, this minimizes the risk of incorrectly identifying non-teeth areas as teeth, which can lead to erroneous diagnoses or treatment plans.

**Recall**: Recall evaluates the proportion of correctly predicted positive pixels to the actual positive pixels in the ground truth, given by:

(9) $$\mathrm{Recall}=\frac{TP}{TP+FN}$$

High recall signifies that the model successfully identifies most of the true positive pixels. This is vital in dental segmentation to ensure that all relevant teeth areas are detected, particularly in cases where some teeth may be partially obscured in the image. A low recall score suggests that the model may overlook significant portions of teeth.

**F1 Score**: The F1 Score combines precision and recall into a single metric, providing a balance between the two:

(10) $$F1\;\mathrm{Score}=2\times \frac{\mathrm{Precision}\times \mathrm{Recall}}{\mathrm{Precision}+\mathrm{Recall}}$$

The F1 Score ranges from 0 to 1, with higher values indicating better segmentation performance. This metric is particularly useful when dealing with imbalanced datasets, as it ensures that both false positives and false negatives are taken into account. A high F1 Score suggests that the model is effective in both detecting teeth and minimizing misclassifications, which is critical for accurate clinical assessments.

In summary, these evaluation metrics provide a comprehensive understanding of the model’s performance by analyzing the alignment of predicted and ground truth pixels. High scores indicate effective segmentation is essential for accurate dental diagnosis and treatment, while low scores highlight areas needing improvement to ensure reliable clinical outcomes.

### 4.3. Quantitative Analysis

Table 2 presents a performance comparison of our model, S2AgScUNet, with several popular deep learning models in the task of dental segmentation, including UNet, SegFormer, MaNet, and CeNet. We evaluate each model’s comprehensive performance using five key metrics: Intersection over Union (IoU), Dice coefficient, Precision, Recall, and F1 Score. Looking at the IoU metric, our model S2AgScUNet (0.8612) outperforms UNet (0.8477) and all other models reviewed, including SegFormer and MaNet. This demonstrates a significant advantage of our model in terms of segmentation accuracy. The Dice coefficient further confirms the effectiveness of S2AgScUNet with a value of 0.9254, indicating a high degree of agreement between the predicted precision and the true annotations. In terms of Precision, S2AgScUNet leads with a score of 0.9239, reflecting our model’s low rate of false positives in identifying dental regions. Additionally, it achieves the highest scores in Recall and F1 Score, at 0.9270 and 0.9254, respectively. A high Recall indicates S2AgScUNet’s strength in minimizing missed detections, while a high F1 Score further validates the model’s excellent balance between Precision and Recall. In summary, S2AgScUNet shows superior performance across all key metrics, particularly in ensuring high precision and recall, which highlights its significant advantages in handling dental segmentation tasks. These results fully demonstrate the potential and practical value of our model in the field of dental image analysis.

Meanwhile, LightUNet, trained using S2AgScUNet as the teacher model, demonstrates highly competitive performance despite significantly reduced parameter count and computational complexity, confirming the effectiveness of our lightweight approach for edge deployment. These results clearly illustrate the potential and practical value of our proposed models in the field of dental image analysis.

**Figure 7:**
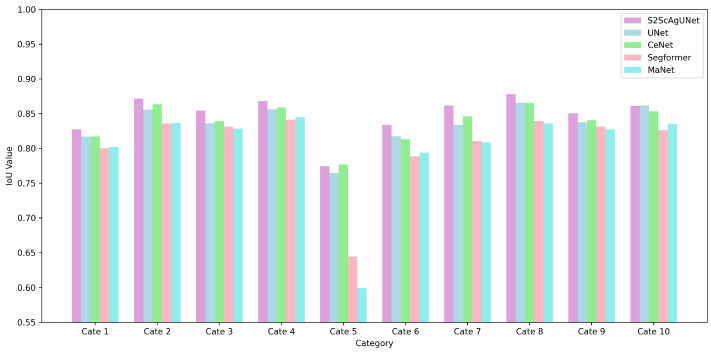
Comparison of IOU across models by category. This figure shows a comparison of IoU scores across ten categories for five models. The S2ScAgUNet model performs best in most of the categories.

Figure 7 presents a comparative analysis of the IoU performance across ten categories for five different models. It is evident from the figure that the S2ScAgUNet model achieves the best performance in all categories compared to other models, such as UNet, CeNet, Segformer, and MaNet. This figure clearly illustrates the robust capability and consistency of the S2ScAgUNet model in handling complex datasets, particularly demonstrating significant advantages in categories 7 and 8.

**Figure 8:**
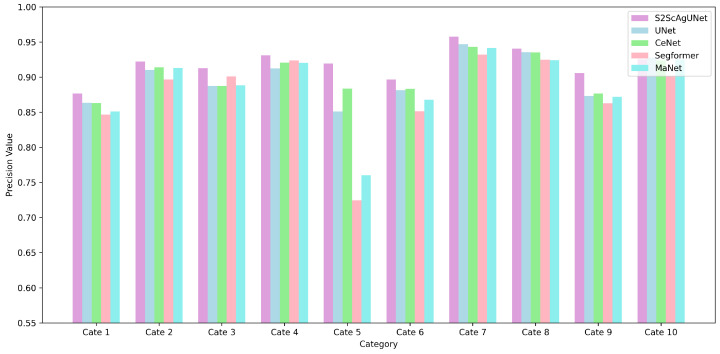
Comparison of precision across models by category. This figure shows a comparison of Precision values across ten categories for five different models. The S2ScAgUNet model consistently achieves the best performance across all ten categories, especially in category 5.

Figure 8 presents a comparative analysis of the Precision performance across ten categories for five different models. It is evident that the S2ScAgUNet model also achieves the best Precision values in most categories, showing significant advantages over other models. Notably, in categories 5 and 7, the Precision of S2ScAgUNet is clearly superior to that of the other models. This result highlights the S2ScAgUNet model’s ability to accurately identify relevant features while minimizing false positives, demonstrating its robustness in handling complex datasets.

### 4.4. Further Analysis

To provide a more intuitive demonstration of the segmentation performance of each model, we conducted visual analyses on representative categories 1, 5, and 8. These visualizations clearly illustrate the performance differences of each model in various scenarios, highlighting their strengths and weaknesses in specific contexts.

**Figure 9:**
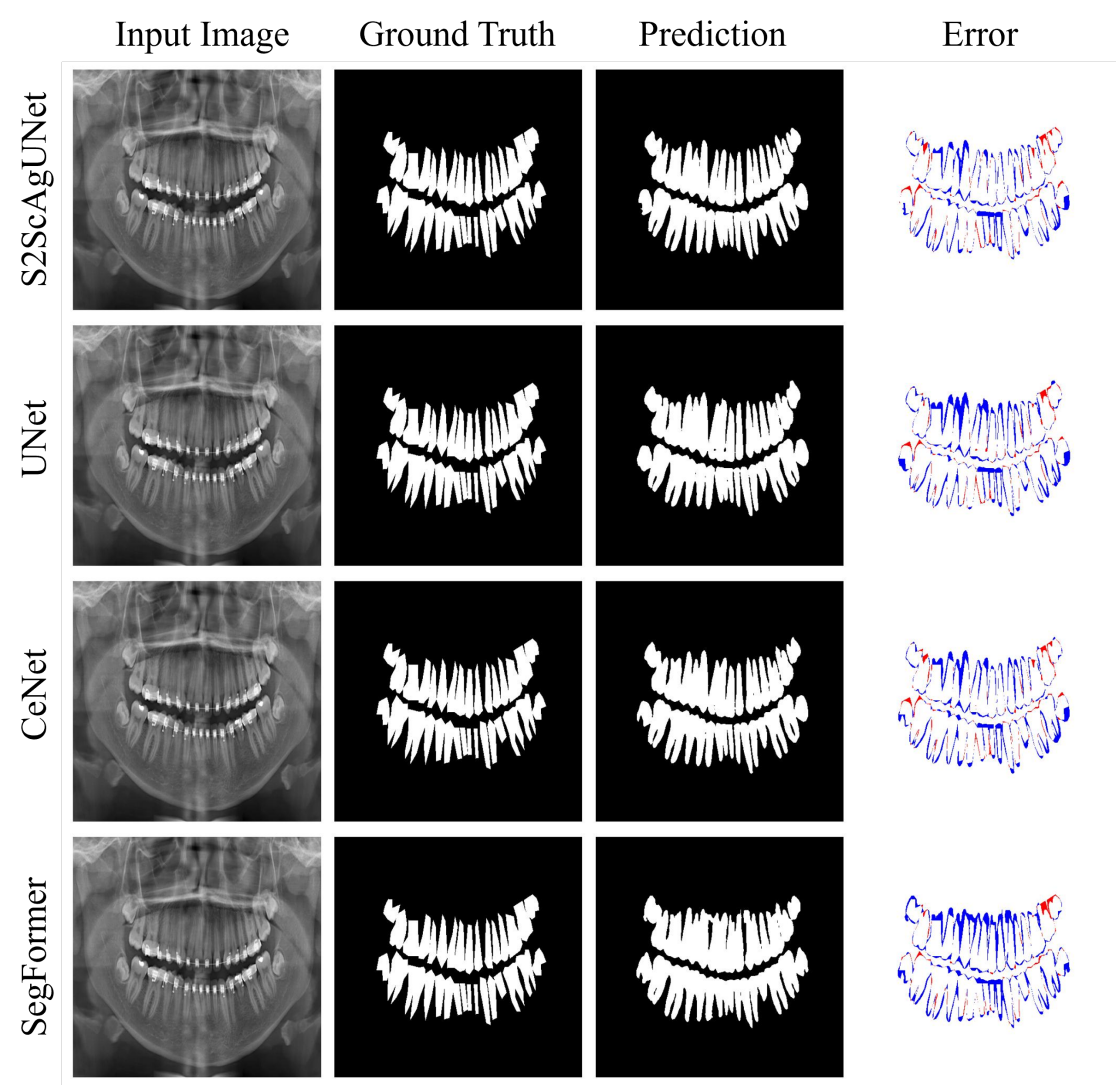
Visual comparison: accuracy and error analysis in tooth segmentation for category 1. The error maps indicate segmentation discrepancies: blue regions represent false positives, while red regions indicate false negatives. The S2ScAgUNet model shows superior performance with fewer errors, demonstrating its effectiveness in accurately capturing tooth boundaries.

Figure 9 displays the results of four different tooth segmentation models (S2ScAgUNet, UNet, CeNet, Segformer) on the same input image. Each row of the figure shows the original input image, the correct annotation (ground truth), the model’s prediction, and the prediction error. A comparative review reveals that the S2ScAgUNet model’s predictions align most closely with the ground truth, with significantly fewer red (indicating missed detections) and blue (indicating false detections) areas in its error image. This demonstrates the S2ScAgUNet model’s higher accuracy and reliability in processing complex dental structures, particularly excelling in capturing fine details at tooth edges. This performance advantage makes the S2ScAgUNet model particularly suitable for dental image processing, especially in clinical applications requiring high-precision tooth identification and segmentation.

**Figure 10:**
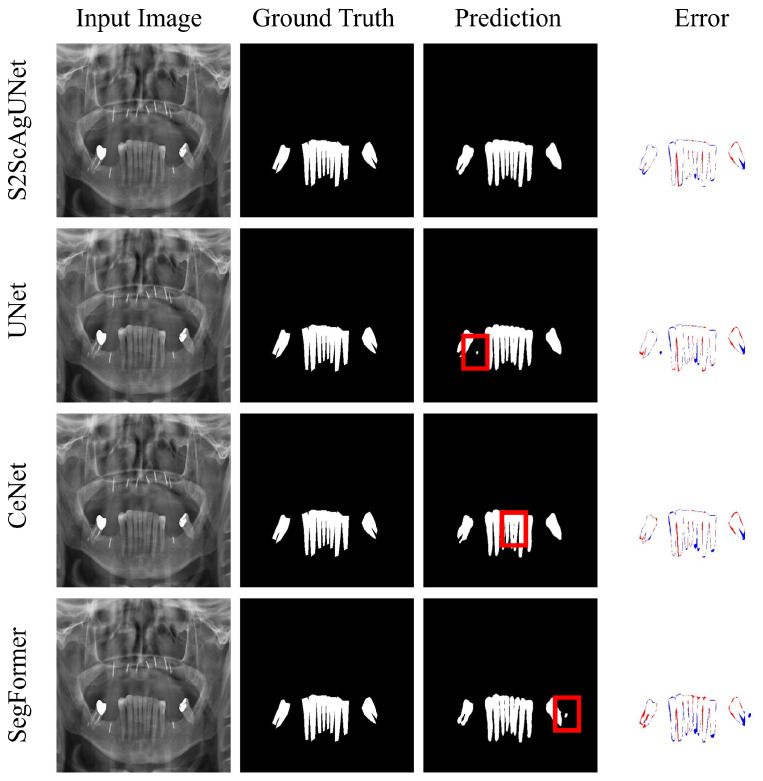
Visual comparison: accuracy and error analysis in tooth segmentation for category 5. The blue areas in the error maps indicate false positives (over-detections), while the red areas indicate false negatives (missed detections). The red boxes highlight regions where the predictions show relatively poor performance. The S2ScAgUNet model outperforms the other models, displaying fewer errors and providing more accurate segmentation in critical regions compared to the highlighted areas in UNet, CeNet, and SegFormer.

Figure 10 illustrates the performance comparison of four dental segmentation models (S2ScAgUNet, UNet, CeNet, Segformer) in Category 5. The error images, marked by red (missed detections) and blue (false detections), clearly highlight that the S2ScAgUNet model aligns most closely with the ground truth and exhibits the least error. In contrast, the other models such as UNet, CeNet, and Segformer show significant errors, particularly in terms of misses and false detections. Notably, the pronounced red markings in the predictions of UNet and Segformer underline the areas these models failed to accurately detect. This visualization underscores the superior accuracy and detail handling of S2ScAgUNet, making it the preferred model for processing similar complex datasets.

The visual analysis of categories 1, 5, and 8 reveals that the S2ScAgUNet model demonstrates superior segmentation performance in most scenarios. Specifically, S2ScAgUNet outperforms other models in capturing details, handling edges, and segmenting complex dental structures, with significantly fewer missed and false detections, which also reflects its strong generalization ability to maintain consistently high accuracy across different categories. In contrast, other models such as UNet, CeNet, and Segformer exhibit certain shortcomings in dealing with complex structures, especially in the edges and finer details of the teeth, where they are prone to missed and false detections. Overall, the S2ScAgUNet model shows greater robustness and generalization in handling high-complexity dental segmentation tasks.

**Figure 11:**
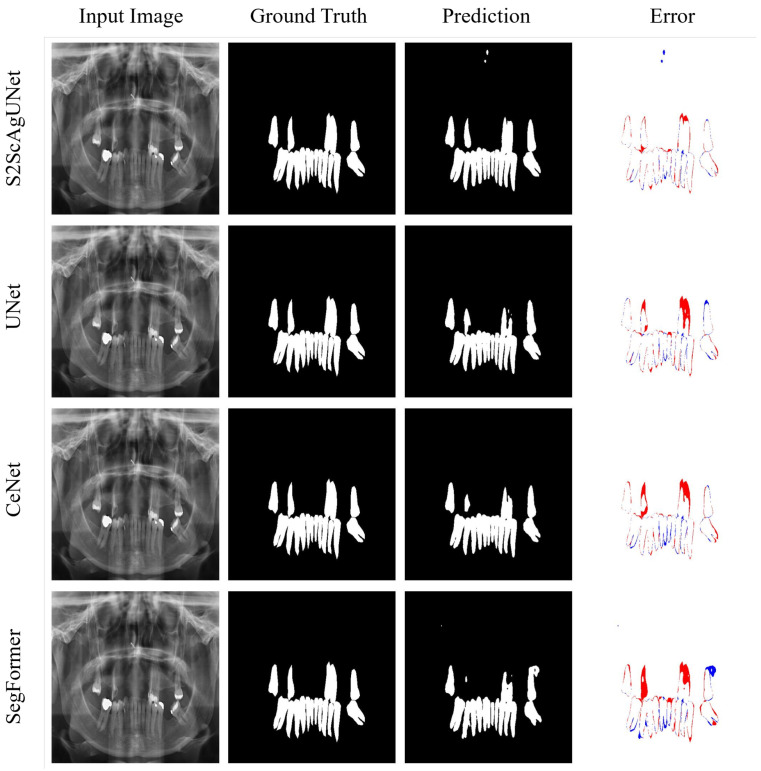
Visual comparison: accuracy and error analysis in tooth segmentation for category 8. Blue indicates false positives, while red indicates false negatives. The figure highlights that the S2ScAgUNet model achieves fewer false negatives compared to UNet, CeNet, and SegFormer.

Figure 11 presents the segmentation results of four dental segmentation models in category 8. From the error maps, it is evident that the S2ScAgUNet model shows significant advantages in segmenting the edges and detailed areas of the teeth. In contrast, the UNet, CeNet, and Segformer models exhibit clear inaccuracies in boundary segmentation, especially in the gaps between teeth and at the tips of the teeth, where missed detections (red areas) and false detections (blue areas) are more pronounced. Specifically, S2ScAgUNet performs best in accurately segmenting the contours and fine structures of each tooth, with almost no visible errors, especially in the detailed segmentation of tooth tips and edges, where its results are nearly identical to the ground truth. In comparison, UNet and CeNet show a tendency to miss some structures at the tops and edges of the teeth, leading to incomplete recognition. Overall, the S2ScAgUNet model demonstrates greater robustness and accuracy in segmenting complex dental structures. It is more effective in capturing tooth details, reducing both missed and false detections.

### 4.5. Ablation Analysis

Table 3 presents the results of the ablation study for the proposed method, showing significant performance improvements across all metrics (IoU, Dice, Precision, Recall, and F1 Score) with the progressive addition of the SAM2, Attention Gate, and ScConvRFB modules. The baseline model has the lowest scores across all metrics. After adding the SAM2 module, there is a notable improvement, which is further enhanced by incorporating the Attention Gate module, strengthening the model’s ability to capture finer details. Finally, with the addition of the ScConv and RFB modules, the model achieves its best performance. This indicates that each module contributes positively to enhancing the segmentation results, with the final configuration significantly boosting the model’s overall performance.

## 5. Conclusions

The proposed S2AgScUNet model effectively improves tooth segmentation performance by integrating the pretrained SAM2 encoder, the ScConv module, and gated attention in skip connections. Experimental results on the UFBA-UESC dataset demonstrate that S2AgScUNet outperforms the traditional UNet model in multiple metrics, including IoU. The introduction of SAM2’s hierarchical feature learning capability significantly enhances the model’s robustness in capturing and segmenting dental details. This is especially evident in small-sample datasets, where it successfully reduces overfitting, showing superior generalization capability. Meanwhile, we used knowledge distillation to achieve performance comparable to the UNet model while reducing the number of parameters to only 490,000, compared to UNet’s 31.05 million, enabling deployment on edge devices.

## Figures and Tables

**Table 1:** Description of the UFBA-UESC dataset.

Category	32 Teeth	Restoration	Dental Appliance	Images	Used Images
1	✓		✓	73	24
2	✓		✓	220	72
3	✓			45	15
4	✓			140	32
5			✓	120	37
6			✓	170	30
7		✓		115	33
8			✓	457	140
9				45	7
10				115	35
Total				1500	425

**Table 2:** Performance comparison among different models. The bold values represent the best performance for each metric. The S2AgScUNet model achieved the best results in all performance metrics. Meanwhile, the lightweight model LightUNet, with significantly reduced parameters and computational complexity, still achieved comparable performance to the UNet model. The parameter count and computational complexity of the S2ScAgUNet model are not comparable due to the use of a large pre-trained model, and therefore, we used “N/A” in the table to represent these metrics. “P”: Params; “F”: FLOPs.

Model	P(M)	F(G)	IOU	Dice	Precision	Recall	F1 Score
UNet	31.05	198.66	0.8477	0.9176	0.9188	0.9164	0.9176
SegFormer	13.68	15.40	0.8196	0.9009	0.8940	0.9079	0.9009
MaNet	35.86	54.18	0.8257	0.9046	0.9074	0.9018	0.9046
CeNet	13.40	126.98	0.8460	0.9165	0.9205	0.9126	0.9165
**S2AgScUNet**	N/A	N/A	**0.8612**	**0.9254**	**0.9239**	**0.9270**	**0.9254**
LightUNet	0.49	3.20	0.8443	0.9156	0.9160	0.9151	0.9156

**Table 3:** Ablation study of the proposed method. The results show the contribution of each module, starting from the baseline model to the addition of SAM2, Attention Gate (AG), and ScConvRFB. Each module contributes to improvements of varying degrees.

Method	IOU	Dice	Precision	Recall	F1 Score
Baseline	0.8477	0.9176	0.9188	0.9164	0.9176
+SAM2	0.8546	0.9216	0.9226	0.9205	0.9216
+AG	0.8559	0.9224	0.9246	0.9201	0.9224
+ScConvRFB	0.8612	0.9254	0.9239	0.9270	0.9254

## Data Availability

All datasets used in this work are publicly available.
